# Influence of age on the relation between body position and noninvasively acquired intracranial pulse waves

**DOI:** 10.1038/s41598-024-55860-6

**Published:** 2024-03-06

**Authors:** Andrea Boraschi, Matthias Hafner, Andreas Spiegelberg, Vartan Kurtcuoglu

**Affiliations:** 1 The Interface Group, Institute of Physiology, University of Zurich, Winterthurerstrasse 190, 8057 Zurich, Switzerland; 2 Zurich Center for Integrative Human Physiology, University of Zurich, Zurich, Switzerland

**Keywords:** Ageing, Neurophysiology, Preclinical research, Biomedical engineering

## Abstract

The capacitive measurement of the head’s dielectric properties has been recently proposed as a noninvasive method for deriving surrogates of craniospinal compliance (CC), a parameter used in the evaluation of space-occupying neurological disorders. With the higher prevalence of such disorders in the older compared to the younger population, data on the head’s dielectric properties of older healthy individuals would be of particularly high value before assessing pathologic changes. However, so far only measurements on young volunteers (< 30 years) were reported. In the present study, we have investigated the capacitively obtained electric signal known as W in older healthy individuals. Thirteen healthy subjects aged > 60 years were included in the study. W was acquired in the resting state (supine horizontal position), and during head-up and head-down tilting. AMP, the peak-to-valley amplitude of W related to cardiac action, was extracted from W. AMP was higher in this older cohort compared to the previously investigated younger one (0°: 5965 ± 1677 arbitrary units (au)). During head-up tilting, AMP decreased (+ 60°: 4446 ± 1620 au, *P* < 0.001), whereas it increased during head-down tilting (− 30°: 7600 ± 2123 au, *P* < 0.001), as also observed in the younger cohort. Our observation that AMP, a metric potentially reflective of CC, is higher in the older compared to the younger cohort aligns with the expected decrease of CC with age. Furthermore, the robustness of AMP is reinforced by the consistent relative changes observed during tilt testing in both cohorts.

## Introduction

Intracranial pressure (ICP) monitoring and craniospinal compliance (CC) assessment are valuable tools for the diagnosis and management of space-occupying disorders of the central nervous system (CNS)^1,2^. ICP and CC are acquired in an invasive manner, which limits their applicability due to inherent risks of complications. To reduce these risks and broaden usability, various approaches for noninvasively measuring ICP or CC have been proposed^3–11^. However, none of them managed to replace the invasive gold standard in routine clinical practice, primarily because they could not provide the required level of reliability and accuracy^12^. Most recently, an approach based on the capacitive measurement of the dielectric properties of the head was introduced^13^. This method has been investigated so far only on young healthy volunteers below the age of 30^14^. Given that ageing affects the cerebrospinal fluid (CSF) system, for example by decreasing ICP^15–17^, it is expected that also the dielectric properties of the head change with age. Testing whether there is such change is the primary aim of this study.

CC is a metric for the change in pressure within the craniospinal compartment (i.e., the space enclosed by the skull, the vertebrae, and the meninges) caused by a change in its volume. CC can be viewed as the sum of the compliances provided by the intracranial and spinal spaces^18,19^. When considering, for example, the intracranial space, the higher the intracranial compliance is, the lower the change in ICP will be with a given change in intracranial volume. Two patients with the same elevated ICP can be at different levels of risk for secondary brain insult depending on their CC, since with low CC minor craniospinal volume variations, e.g., due to bleeding, can provoke a life-threatening increase in ICP^20^.

The capacitive acquisition of the dielectric properties of the head has recently been suggested as a noninvasive technique for deriving CC surrogates^13,21^. By employing two electrically isolated capacitor electrodes placed on the scalp, an electric signal termed W is obtained from the voltage amplitude of the LC-oscillator circuit formed by an inductor within the measurement device, the isolated electrodes and the subject’s head^13^. The W signal exhibits characteristic oscillations with the same frequencies as respiration and cardiac action. These oscillations are attributed to periodic variations of CSF and blood volumes in the head that occur with each cardiac and respiratory cycle and cause transient changes to the dielectric properties of the head^11^. It has been shown through hyperventilation testing on healthy volunteers that W is, at least in part, of intracranial origin^13^. The peak-to-valley amplitude (AMP) of W associated with cardiovascular activity has been shown to decrease with head-up tilting and increase with head-down tilting in healthy subjects below the age of 30^14^. Tilt testing is a noninvasive maneuver known to change the relative contributions of the cranial and spinal compartments to CC^19,22^.

Space-occupying disorders reduce CC, and measurements of CC can be used for the diagnosis of such. The incidence and prevalence of space-occupying disorders increase with age. For example, van Asch et al. reported an increase in the incidence ratio of intracerebral hemorrhage with age, from 0.10 in people below 45 years to 9.6 in subjects older than 85 years (using the age group of 45–54 years as reference)^23^. Isaacs et al. reported a mean prevalence of hydrocephalus of 175 cases per 100,000 individuals aged 65 years or older, compared to 11 cases in the adult (19–64 years) and 88 cases in the pediatric (≤ 18 years) population^24^.

Normal aging can have effects comparable to those produced by pathologies. For instance, arterial stiffness increases with age^25^ due to changes in the elastin and collagen composition of the vascular tissue^26,27^. This translates to increased systolic blood pressure and pulse pressure (difference between systolic and diastolic blood pressure), and increased pulse wave velocity^28^. CSF flow dynamics also change with age^29^. ICP decreases with age^15–17^, presumably due to reduced CSF production^30^. At the same time, CSF outflow resistance increases^31^. Intracranial compliance is expected to decrease with age^32–34^, with a significant association observed between brain elastance (the inverse of intracranial compliance) and frailty^35,36^. Conversely, the contribution of intracranial compliance to CC was shown to increase with age^17^.

It is reasonable to postulate that changes to the cardiovascular and CSF systems with age will affect the head’s dielectric properties and, thereby, also the noninvasively measured signal W. Therefore, before evaluating changes to potential CC surrogates derived from W with respect to pathologic causes, the effects of aging must be understood. In this study, we acquired W in a cohort of healthy volunteers above 60 years of age and measured AMP, a metric potentially linked with CC, during tilt testing. We then compared the obtained AMP values and their change during tilting with those observed in a previously investigated cohort of younger (< 30 years) healthy subjects^14^.

## Methods

### Study design

**Figure 1:**
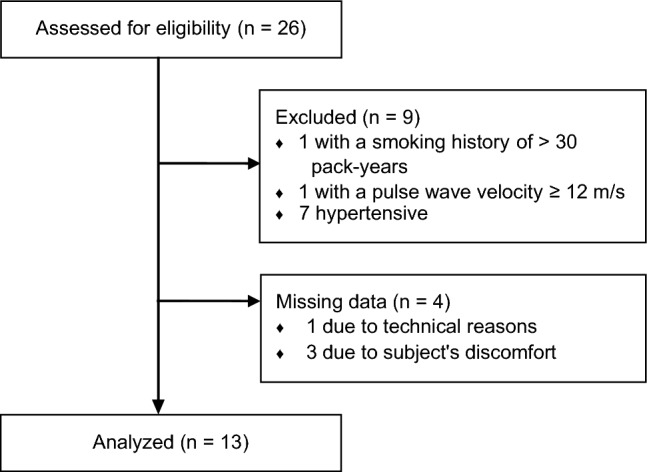
CONSORT flow diagram of the study.

The only inclusion criterion was age > 60 years. Subjects were excluded if they presented with neurological, neuromuscular or craniospinal pathologies; a medical history with prior cranial or spinal surgical interventions; any implant or non-removable foreign object in head or neck; cardiac or pulmonary pathologies; a smoking history with > 30 pack-years^37,38^; or a pulse wave velocity ≥ 12 m/s^39^ as assessed from the time difference between pulse waves acquired, at protocol start, at the right index finger and at the ipsilateral second toe (pOpmètre 300s, Axelife SAS, Saint-Nicolas-de-Redon, France). Participants were asked to refrain from consuming tobacco, food, alcoholic and caffeinated beverages in the three hours prior to the physiological tests. Over the course of the experimental session, brachial arterial blood pressure (ABP) was measured twice by oscillometry (Finapres Nova, Finapres Medical Systems B.V., Amsterdam, The Netherlands), with the two acquisitions performed approximately 1 h apart. A systolic ABP ≥ 140 mmHg and/or a diastolic ABP ≥ 90 mmHg at both was a further exclusion criterion, this based on the definition of hypertension by the World Health Organization^40^. 26 subjects were assessed for eligibility. Of these, 9 were excluded based on the aforementioned criteria. In particular, 7 of the excluded 9 subjects (27% of the study population) exhibited levels of ABP satisfying the definition of hypertension by the World Health Organization^40^. Their exclusion is in line with the study aim to assess the effect of physiological aging on W signal features. Of the collected datasets, 4 were not further considered because the measurements at one or more tilt angles could not be performed due to technical reasons (1 dataset) or discomfort experienced by the respective test person (3 datasets). To allow for proper comparison of the data collected on the current, older study participants with those of the previous, younger cohort^14^, we employed the same study protocol, which required each subject to spend 2.5 h overall on the tilt table. While this was well tolerated by the younger subjects, the excluded 3 older volunteers experienced a level of discomfort that required interruption of the tests. Ultimately, 13 individuals (6 women) were included in the final analysis (age: 68 ± 4 years), as detailed in the CONSORT flow diagram in Fig. 1. A description of the study cohort is provided in Table 1.

**Table 1:** Characteristics of the current study cohort compared to a previously investigated^14^ cohort of younger volunteers.

	Older cohort (n = 13)	Younger cohort^14^ (n = 18)
Age	68 ± 4	25 ± 2***
Height (cm)	169 ± 10	170 ± 10
Weight (kg)	69 ± 13	68 ± 12
Head circumference (cm)	58 ± 2	56 ± 2
PWV (m/s)	8 ± 2	5 ± 1***

Mean ± SD are reported. PWV: pulse wave velocity. Asterisks indicate statistically significant differences between the older and younger cohorts. *** *P* < 0.001.

### Measurement device for noninvasive acquisition of the head’s dielectric properties

Two electrically isolated electrodes (PEM2, Cephalotec, Horgen, Switzerland) connect the device for noninvasive acquisition of the head’s dielectric properties (PEM1, Cephalotec) to the subject’s head. The measurement method is capacitive in nature, since the copper electrodes are electrically isolated from the scalp by a thin polyurethane layer. The device-electrode-head circuit is excited by a high frequency (~ 1 MHz) carrier wave with constant current amplitude. Changes in the head’s composition due to variations in blood and CSF volumes during cardiac and respiratory cycles modulate the head’s dielectric properties, which, in turn, modulate the voltage amplitude between the electrodes. Through signal processing operations (amplitude demodulation; bandpass filtering with a passband from 0.1 to 4.9 Hz, to preserve only the frequency range relevant for cardiac and respiratory activities; notch filtering to eliminate the power line interference; amplification and A/D conversion), the signal W is obtained, characterized by cardiac and respiratory modulation. We refer the reader to Spiegelberg et al.^13^ for more detailed information on the working principle of the measurement device and on the relationship between W and the dielectric properties of the head.

### Protocol

**Figure 2:**
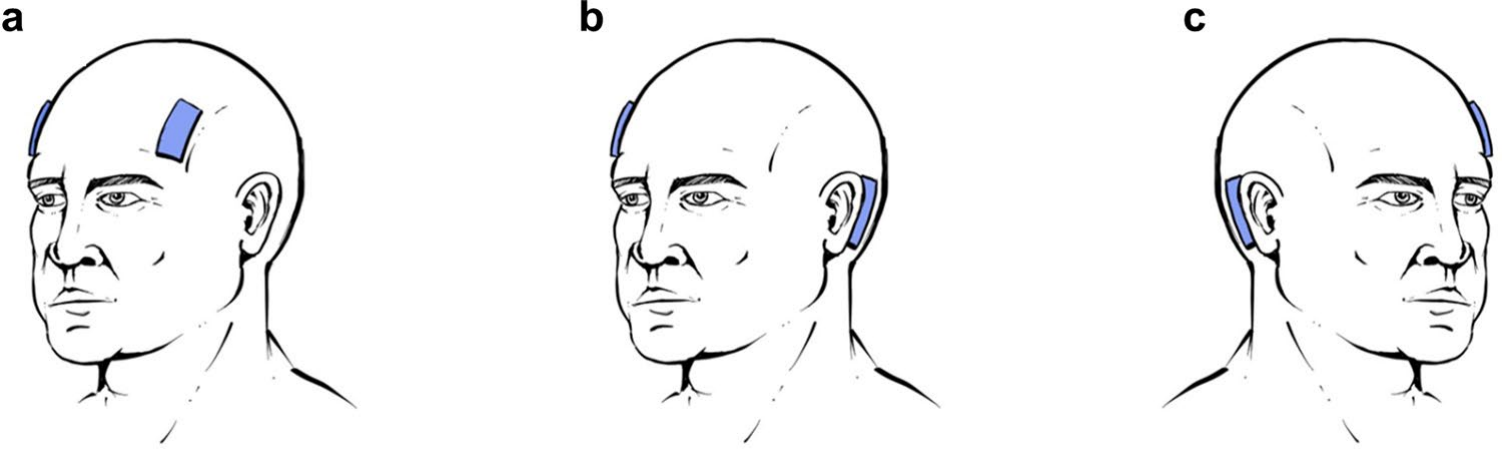
Isolated electrodes (blue) placed in regions corresponding, in a 10–20 electroencephalogram setup, to: (**a**) the F3, F7 and F4, F8 electrodes (*forehead positioning*); (**b**) the F4, F8 and Cb1 electrodes (*cross-positioning, right top*); (**c**) the F3, F7 and Cb2 electrodes (*cross-positioning, left top*).

After measurements of height, weight and head circumference, subjects lay down supine on a tilt table in horizontal position (0°). The electrical activity of the heart was measured by three-lead electrocardiography (ECG). ABP was acquired continuously and noninvasively through a photoplethysmographic finger cuff wrapped around the middle finger’s second phalanx on the left hand (Finapres Nova). Correction of the obtained ABP waveform to the heart level was performed using a height sensor secured at the fourth intercostal space. Furthermore, the ABP waveform was calibrated using two measurements of systolic and diastolic brachial ABP, acquired by oscillometry via an arm cuff following the automated calibration protocol of the Finapres Nova. The same measurements were used to exclude subjects suspected of hypertension. To measure W, two electrically isolated electrodes were placed symmetrically on the subject’s forehead in areas corresponding to, respectively, the F3, F7 and F4, F8 electrodes in a 10–20 electroencephalogram setup^41^ (Fig. 2a). We refer to this electrode arrangement as *forehead positioning*. After approximately 10 min at 0° to let the subjects reach a steady-state baseline condition, the tilt table was moved to + 10°, + 30°, + 45°, + 60°, 0° (control), − 15° and − 30° (positive angles correspond to head-up tilting, HUT; negative angles to head-down tilting, HDT). At 0°, + 60° and − 30°, W was acquired also with electrodes placed in areas corresponding either to the F4, F8 and Cb1 electrodes, or to the F3, F7 and Cb2 electrodes, in a 10–20 electroencephalogram setup^41^ (Fig. 2b,c, respectively). Respectively, these electrode placements are referred to as *cross-positioning, right top*, and *cross-positioning, left top*. Between *cross-positioning, right top* and *cross-positioning, left top*, the configuration easier to place was chosen, primarily based on the individual’s hair cover. This was done assuming that *cross-positioning, right top* and *cross-positioning, left top* are equivalent because of symmetry, which was confirmed by statistical testing. Starting from *forehead positioning*, an additional electrode was applied to obtain *cross-positioning*. This allowed for the acquisition configuration to be switched from *forehead* to *cross-positioning* by simply reconnecting the measurement device without having to reposition or otherwise move the electrodes. At each angle and for each electrode configuration, the measurement device was re-started, requiring about 3 min for the internal initialization procedure, which also allowed the subject to regain a steady state. Afterwards, signals were acquired for 2 min.

### Data analysis

**Figure 3:**
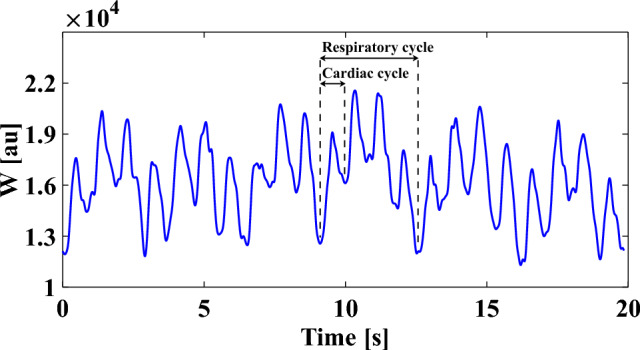
Illustrative 20 s time window of W acquired on a volunteer. Signal oscillations due to cardiac and respiratory action can be observed.

The signals were recorded with a sampling frequency of 100 Hz through a USB data acquisition box (DT9804, Measurement Computing Corp., Norton, Massachusetts, USA) on a laptop equipped with ICM + software (Cambridge Enterprise, Cambridge, UK) and synchronized prior to analysis. The synchronization was performed based on time delay values reported by the manufacturers of the respective equipment in the signal chain. Heart rate (HR) was determined in real-time by the Finapres Nova, from either the ECG signal or the ABP waveform. Mean arterial blood pressure (MAP) and pulse pressure (PP) were computed offline from the continuously and noninvasively measured ABP waveform. In all subjects, W exhibited characteristic modulation induced by cardiac and respiratory activity (Fig. 3).

**Figure 4:**
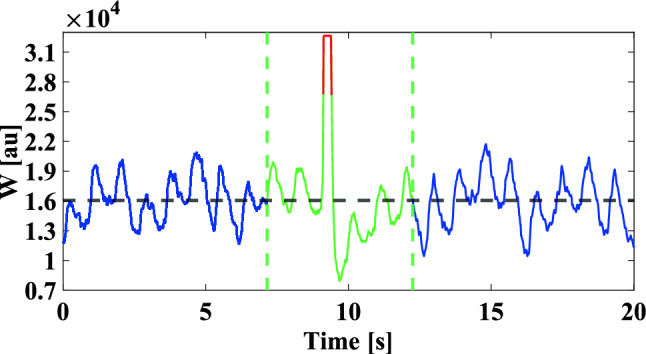
Example of an artifact in the W signal. Both the outliers (highlighted in red) as well as data points five periods before and after them (highlighted in green) were excluded from analysis (see text for definition of period).

Artifacts in W due to sudden head movements, touching of the isolated electrodes, or vigorous swallowing were removed from W using a median-based approach: Considering the entire W dataset for a given tilt angle, data points were classified as outliers if they were 3 interquartile ranges below the 25th percentile or above the 75th percentile. Outliers as well as all data points five periods before and after each outlier were excluded from analysis, where a period is the time interval between the outlier and the first passage of W through its median value (first period), or between two consecutive passages of W through its median value (following periods). An example of an artifact is shown in Fig. 4. In the remaining data, the peak-to-valley amplitude (AMP) of W related to cardiac activity was computed as the difference between the values of W at systole (peaks) and diastole (valleys). As reference for peak detection in the W signal, the R-peaks of the concomitantly acquired lead-II ECG signal were employed.

All parameters were calculated over a 1 min steady-state time window obtained at each tilt angle. Steady state was presumed if, for all analyzed metrics, the respective median in all 10 s frames within the examined 1 min did not deviate by more than 20% from the corresponding median in the first 10 s window.

### Statistics

Unless otherwise specified, data are reported as mean ± standard deviation (SD). A Shapiro-Wilk test was used to verify normality. After checking that for all variables there was no statistically significant difference between the two measurements at 0° (either with a paired t-test if the data were normal, or with a Wilcoxon signed-rank test if not), the average of the measurements at 0° was employed as reference. For variables showing normal data distributions at each tilt angle, values at different tilt angles were compared to the 0° reference condition by paired t-test, applying Holm correction^42^. Logarithmic transformation was carried out for variables that showed not normally distributed data at one or more tilt angles. If the log-transformed data did not show normal distribution as well, values at different tilt angles were compared to the 0° reference condition by Wilcoxon signed-rank test, applying Holm correction. When performing the Holm correction, the statistical tests carried out in the previous study on younger volunteers^14^ were not considered. We deemed *P*-values < 0.05 to be indicative of statistically significant differences.

### Ethics approval and consent to participate

This study was approved by the Ethics Committee of the Canton of Zurich (BASEC-Nr. 2018-02078). The study was conducted in accordance with the principles outlined in the Declaration of Helsinki and in accordance with local statutory requirements. All participants provided written informed consent.

## Results

### Hemodynamic parameters

To ensure the adequacy of our tilt testing protocol, and to aid in the interpretation of the AMP data, HR, MAP and PP were acquired. These were compared to published values and used to assess whether changes related to the cardiovascular system, such as variations in the stroke volume (SV) of the heart, may have contributed to changes in AMP.

HR rose as of a tilt angle of + 45° during HUT (0°: 58 ± 8 beats/min; + 10°: 56 ± 7 beats/min, *P* = 0.231; + 30°: 60 ± 9 beats/min, *P* = 0.1; + 45°: 64 ± 10 beats/min, *P* = 0.04; + 60°: 70 ± 12 beats/min, *P* = 0.003). HR returned to its baseline value during the control acquisition (0°: 56 ± 8 beats/min, *P* = 0.074), and remained stable during HDT (− 15°: 56 ± 7 beats/min, *P* = 0.89; − 30°: 58 ± 8 beats/min, *P* = 0.89). HUT provoked no significant change in MAP. During HDT, MAP increased (0°: 98 ± 8 mmHg; − 15°: 106 ± 12 mmHg, *P* = 0.005; − 30°: 113 ± 12 mmHg, *P* < 0.001). PP decreased at a HUT angle of + 60° (0°: 54 ± 8 mmHg; + 10°: 56 ± 9 mmHg, *P* = 0.824; + 30°: 53 ± 9 mmHg, *P* = 0.23; + 45°: 52 ± 8 mmHg, *P* = 0.23; + 60°: 45 ± 6 mmHg, *P* = 0.003). After returning to its baseline value during the control acquisition (0°: 57 ± 11 mmHg, *P* = 0.078), PP remained stable during HDT (− 15°: 59 ± 15 mmHg, *P* = 0.705; − 30°: 57 ± 14 mmHg, *P* = 0.824). Values of HR, MAP and PP are reported in Table 2.

**Table 2:** Hemodynamic parameters and peak-to-valley amplitude of the W signal associated with cardiac action (AMP) at each tilt angle. Acquisition with electrodes in *forehead positioning.*

	0°	+ 10°	+ 30°	+ 45°	+ 60°	0°	− 15°	− 30°	0° ref
HR [beats/min]	58 ± 8	56 ± 7	60 ± 9	64 ± 10*	70 ± 12**	56 ± 8	56 ± 7	58 ± 8	57 ± 8
MAP [mmHg]	98 ± 8	99 ± 12	99 ± 12	102 ± 13	101 ± 13	102 ± 13	106 ± 12**	113 ± 12***	100 ± 10
PP [mmHg]	54 ± 8	56 ± 9	53 ± 9	52 ± 8	45 ± 6**	57 ± 11	59 ± 15	57 ± 14	55 ± 9
AMP [au]	5965 ± 1677	4856 ± 1208**	4819 ± 1148**	4635 ± 1348***	4446 ± 1620***	5725 ± 2118	7116 ± 2153***	7600 ± 2123***	5845 ± 1752

Mean ± SD are reported. 0° reference (*0° ref*) indicates the average of the two 0° values. HR: heart rate, MAP: mean arterial pressure, PP: pulse pressure, AMP: peak-to-valley amplitude of W associated with cardiac action. Statistically significant differences from the respective 0° reference are marked with asterisks. * *P* < 0.05, ** *P* < 0.01, *** *P* < 0.001.

### Peak-to-valley amplitude of the W signal associated with cardiac action (AMP)

To determine whether the placement location of the isolated electrodes affects AMP, values obtained with *cross-positioning* of the electrodes (Fig. 2b,c) were compared to those acquired with the established *forehead positioning* (Fig. 2a). In addition to *forehead positioning*, AMP was acquired in 5 subjects with the *cross-positioning, right top* setup. *Cross-positioning, left top* was used for the remaining 8 subjects. There were no statistically significant differences in AMP between these two configurations at any tilt angle. Therefore, AMP values acquired with the isolated electrodes in *cross-positioning, right top* or in *cross-positioning, left top* are presented in Table 3 in aggregated form.

**Table 3:** Hemodynamic parameters and peak-to-valley amplitude of the W signal associated with cardiac action (AMP) at each tilt angle. Acquisition with electrodes in *cross-positioning*.

	0°	+ 60°	0°	− 30°	0° ref
HR [beats/min]	58 ± 9	69 ± 12*	57 ± 9	59 ± 9	57 ± 9
MAP [mmHg]	101 ± 9	102 ± 14	100 ± 14	116 ± 13***	101 ± 11
PP [mmHg]	55 ± 8	47 ± 7*	55 ± 9	58 ± 9	55 ± 8
AMP [au]	5638 ± 1302	4114 ± 1201**	5432 ± 1728	6758 ± 1518***	5535 ± 1490

Mean ± SD are reported. 0° reference (*0° ref*) indicates the average of the two 0° values. HR: heart rate, MAP: mean arterial pressure, PP: pulse pressure, AMP: peak-to-valley amplitude of W associated with cardiac action. Statistically significant differences from the respective 0° reference are marked with asterisks. * *P* < 0.05, ** *P* < 0.01, *** *P* < 0.001.

**Figure 5:**
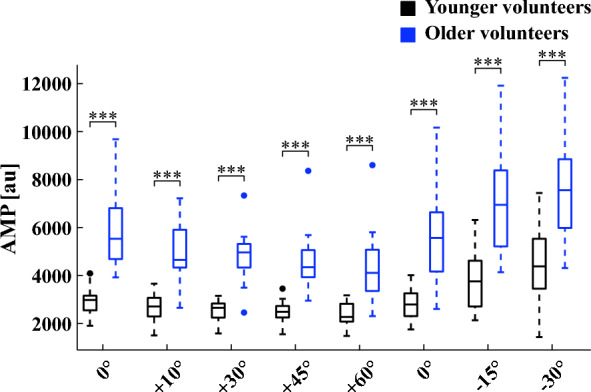
Boxplots of AMP, the peak-to-valley amplitude of W associated with cardiac activity, during tilting in the younger (< 30 years) and older (> 60 years) healthy volunteer cohorts. The central line in each box indicates the median, while the bottom and top horizontal edges refer to the 25th and 75th percentiles, respectively. Data points 1.5 interquartile range below the 25th percentile, or 1.5 interquartile range above the 75th percentile are marked with full circles. Bottom and top whiskers extend to the lowest and highest values when data points marked with full circles are not considered. Statistically significant differences between younger and older healthy volunteer cohorts at each tilt angle are marked with asterisks. *** *P* < 0.001.

To assess possible effects of aging on the dielectric properties of the head, AMP was acquired during tilt testing and compared to values previously obtained on younger healthy volunteers below 30 years of age (n = 18)^14^. With the isolated electrodes in *forehead positioning*, AMP diminished during HUT (0°: 5965 ± 1677 arbitrary units (au); + 10°: 4856 ± 1208 au, *P* = 0.002; + 30°: 4819 ± 1148 au, *P* = 0.002; + 45°: 4635 ± 1348 au, *P* < 0.001; + 60°: 4446 ± 1620 au, *P* < 0.001). AMP returned to its baseline value during the control acquisition (0°: 5725 ± 2118 au, *P* = 0.393), and increased during HDT (− 15°: 7116 ± 2153 au, *P* < 0.001; -30°: 7600 ± 2123 au, *P* < 0.001). Values of AMP acquired with the isolated electrodes in *forehead positioning* are reported in Table 2 and in Fig. 5. With the electrodes in *cross-positioning*, AMP decreased at + 60° (0°: 5638 ± 1302 au; + 60°: 4114 ± 1201 au, *P* = 0.001). After returning to its baseline value during the control acquisition (0°: 5432 ± 1728 au, *P* = 0.373), AMP increased with HDT (− 30°: 6758 ± 1518 au, *P* < 0.001). Values of AMP acquired with the isolated electrodes in *cross-positioning* are reported in Table 3.

## Discussion

We have investigated the difference in the head’s dielectric properties between two cohorts of healthy individuals: one over 60 years of age, and one below 30 years. Both cohorts were tested with the same measurement instrumentation, with the same protocol and by the same team, and the collected data were post-processed with the same algorithms, as reported here and in Boraschi et al.^14^. In particular, we measured capacitively the electric signal W previously shown to be, partially, of intracranial origin^13^. We then analyzed AMP, the amplitude of the oscillation of W caused by cardiovascular action, which may be reflective of craniospinal compliance (CC)^14^. The older cohort showed significantly higher AMP in horizontal supine position and during head-up and head-down tilting. Given that CC is expected to decrease with age^32–34^, this suggests that age-related differences in CC may be reflected in W.

There was no significant difference between the two cohorts in terms of body height, weight, and head circumference (Table 1). The older group presented with higher PWV, with the measured values aligning with those reported in^43^ for both age groups. This is a reflection of typical physiologic increase in arterial stiffness with age, which leads to reduced compliance of the aorta and large arteries that provide Windkessel function^25^. We also observed the expected trend towards higher PP in the older cohort, but the difference between the two groups was not significant (*0° ref*, older: 55 ± 9 mmHg, younger: 52 ± 9 mmHg, *P* = 0.37). We attribute this to the limited sample size, as the mean PP difference of 3 mmHg between the younger and older cohort is comparable to what Chou et al. have reported^44^. We saw no significant difference in HR (*0°* *ref*, older: 57 ± 8 beats/min, younger: 61 ± 10 beats/min, *P* = 0.535), which is in agreement with the results from^45,46^, though it is debated whether and how HR changes with age in healthy subjects^47^.

In the horizontal reference position, the older cohort exhibited a significantly higher AMP with electrodes in *forehead positioning* (*0° ref*, older: 5845 ± 1752 au, younger: 2869 ± 597 au, *P* < 0.001). Considering that height, weight, and head circumference were not different (Table 1), the higher AMP in the older group cannot be ascribed to these factors. It is conceivable that a difference in cardiac SV would also change AMP. However, given that there was no difference in HR and in static body measures, it is unlikely that the cardiac SV differed between the two groups. This is supported by the cardiac SV estimate provided by the Finapres Nova using a statistical model of the cardiovascular system^48^ (*0° ref*, older: 92 ± 21 mL, younger: 81 ± 17 mL, *P* = 0.183). We note that the AMP SD was higher in the older cohort. This is not unexpected for two reasons: Firstly, aging affects neurological and cardiovascular regulatory mechanisms^49^, leading to an increase in physiologic heterogeneity with age^50^. Secondly, the prevalence of pathologies in general increases with age. While our exclusion criteria were selected to minimize confounders, it is possible that pathologies not captured by the criteria may have contributed to the higher variability in the older group. As a case in point, removing the data of one subject each with the highest contribution to the variability to their respective groups reduces the difference in SD between the two groups at *0° ref* from 9 to 5 percentage points.

The higher AMP in the older cohort may be a consequence of a larger cervical CSF SV, i.e., of more CSF being exchanged with each cardiac cycle between cranial and spinal compartments^51^. This may be a consequence of reduced intracranial compliance with age, as observed in patients with symptoms of hydrocephalus^32^, and in traumatic brain injury patients^33,34^. To our knowledge, the relationship between invasively assessed CC and age has not been studied in healthy individuals. Considering no difference in cardiac SV, an increase in cervical CSF SV will increase the amplitude of oscillation of intracranial blood-to-CSF ratio, and thus also AMP^14^. This interpretation is supported by finite element electromagnetics simulations^11,14^, and by Lokossou and colleagues who reported higher cervical CSF SV in an older (age: 73 ± 6 years) compared to a younger (age: 31 ± 7 years) healthy cohort^51^. Given the significant positive correlation between aqueductal and cervical CSF SV^51^, our interpretation of the AMP data is also supported by the study of Sartoretti et al. who observed a higher aqueductal CSF SV with age^52^. However, we must point out that there is no agreement in the literature on the course of cervical CSF SV as we get older: Burman and colleagues reported a decrease, rather than an increase, of this metric with age, though we note that no cervical CSF SV data were provided for the age range included in the present study (i.e., > 60 years)^17^. Stoquart-ElSankari et al., too, observed a reduction of cervical CSF SV with age^53^. However, the sex ratio differed between the younger and older cohorts studied, with male-to-female ratio of 16:3 (younger) and 4:8 (older), respectively^53^. Since sex influences CSF SV (higher for males)^29,52^, the higher proportion of males in the younger group may have confounded the observed decrease in SV.

Tilting elicited cardiovascular responses in both cohorts. During HUT, a baroreflex-mediated increase in HR was observed, which compensates for the reduction in cardiac SV (both right and left ventricular SV) due to the pooling of venous blood in the lower body^54^ and the consequent reduction in venous return^55^. Such increase in HR occurs primarily via a decrease in vagal tone^56^, and after roughly 30 s from tilting, an increase in total peripheral vascular resistance is also observed^57^. Notably, but as expected^58^, the HR response to HUT was attenuated in the older group compared to what we had previously observed in the younger (change from *0° ref* to + 60°, older: + 13 ± 10 beats/min, younger: + 29 ± 13 beats/min, *P* < 0.001). Despite this, MAP was maintained at all HUT angles. In addition to the baroreflex mediated by the carotid baroreceptors, an intracranial baroreflex triggered by the increase in ICP^59,60^ may also play a role during HDT^61^. In the older cohort, HDT did not affect HR and PP, but MAP increased. At the investigated HDT angles, an MAP increase is not expected, but it is consistent with what Cunningham et al. reported for larger HDT angles^62^. These observations corroborate the adequacy of the employed tilting protocol.

Tilting alters the relative contributions of the spinal and cranial compartments to CC, for example decreasing intracranial compliance and increasing spinal compliance during HDT. Therefore, AMP is expected to be higher in head-down compared to normal supine position^14^, since it depends primarily on the oscillations of intracranial blood and CSF volumes. Indeed, AMP increased with HDT and decreased with HUT in both groups. In our previous study^14^, we attributed this change in the younger group to a change in cervical CSF SV with body position^63,64^, and we propose the same for the older group, as further supported by data from electromagnetics simulations^11,14^. During HUT, the reductions of AMP at + 10° and + 30° in the older group are of particular interest, because they are not accompanied by changes in the measured cardiovascular parameters. This further supports our interpretation that primarily changes in CSF SV rather than cardiovascular factors, which may not be reflective of altered CC, are responsible for the positional variation of AMP. Furthermore, the decrease in AMP observed between 0° and + 10° was larger than the subsequent reductions during HUT. This may be due to the collapse of the internal jugular veins^65^. Indeed, this protective mechanism, which engages during HUT beyond + 20°^66^, limits ICP reduction in upright position and modulates CSF volume and compliance shifts during HUT^22^, constraining the reduction of AMP. A similar pattern in AMP was also observed in younger volunteers^14^.

AMP showed similar behavior during tilting when W was acquired with forehead or cross-positioning of the electrodes. This supports our assumption that even though W is influenced by electrode positioning, the contribution of cyclic fluid shifts, which depend on compliance, is sufficiently high to yield a robust signal. We note that in our previous study with the younger cohort, the influence of electrode position was not investigated. Therefore, a comparison of corresponding results between the age groups could not be carried out.

We note that while there were marked differences in AMP between the younger and older cohorts, substantially larger sample sizes would be needed to establish age-dependent reference values. Moreover, due to technical limitations, the W frequency range between 0 and 0.1 Hz, which might include physiologically relevant features such as those associated with B-waves^67^, could not be acquired. We further note that W reflects the dielectric properties of the entire head, and not only of the intracranial space. The relative intensities of the intra- and extracranial components of W are currently unknown. Finally, changes in the intracranial blood volume might also affect W. To quantify the impact of these potential confounding factors on CC surrogate candidates, invasive acquisition of CC using CSF tap or infusion testing with concomitant measurement of W is required.

## Conclusions

We investigated the effect of age on AMP, a metric potentially reflective of CC derived from the noninvasively measured electric signal W. AMP was higher in the older cohort (> 60 years of age) compared to the younger (< 30 years), which is in agreement with the expected lower CC in the older group. The two cohorts showed the same relative behavior of AMP during tilt testing, and changing electrode positions did not affect this behavior, both of which speak for the robustness of this metric. Further investigations are warranted to confirm a potential association between AMP and invasively measured CC.

## Data Availability

The data supporting the conclusions of this study are available upon reasonable request from the corresponding author after approval by the Ethics Committee of the Canton of Zurich.
